# Pretreatment Claudin-18.2 Expression Predicts Poorer Survival Outcomes in Locally Advanced Gastric Cancer Treated with Perioperative Chemotherapy

**DOI:** 10.3390/diagnostics16091277

**Published:** 2026-04-23

**Authors:** Gürkan Gül, Özlem Kutlu, Asuman Argon, Halil Taşkaynatan, Özlem Özdemir

**Affiliations:** 1 Department of Medical Oncology, Izmir City Hospital, Izmir 35540, Turkey; ozlemacikerkutlu@gmail.com (Ö.K.); haliltaskaynatan@gmail.com (H.T.); ozdemirozlem.md@gmail.com (Ö.Ö.); 2 Department of Pathology, Izmir City Hospital, Izmir 35540, Turkey; asumanargon@gmail.com

**Keywords:** gastric cancer, CLDN18.2, neoadjuvant chemotherapy, immunohistochemistry, prognostic biomarker, relapse-free survival, FGFR2b

## Abstract

**Background/Objectives**: Claudin-18.2 (CLDN18.2) has recently emerged as a therapeutic target in gastric cancer; however, its prognostic relevance in the neoadjuvant setting remains insufficiently defined. We evaluated the clinical significance of CLDN18.2 and fibroblast growth factor receptor 2b (FGFR2b) expression in patients with locally advanced gastric cancer treated with neoadjuvant therapy. **Methods**: This retrospective single-center study included 64 patients with locally advanced gastric cancer who received neoadjuvant chemotherapy followed by curative surgery. Pretreatment endoscopic biopsy specimens were analyzed using immunohistochemistry to assess CLDN18.2 and FGFR2b expression. Survival outcomes were evaluated using Kaplan–Meier analysis and Cox proportional hazards regression models. **Results**: CLDN18.2 positivity was detected in 29.7% of patients and was not associated with baseline clinicopathological characteristics or pathological treatment response. However, CLDN18.2-positive tumors demonstrated significantly shorter relapse-free survival (median 19.0 vs. 36.6 months, *p* = 0.038) and overall survival (median 28.9 vs. 53.4 months, *p* = 0.005). In multivariable analysis, CLDN18.2 positivity remained an independent predictor of relapse-free survival. FGFR2b positivity was observed in 14.1% of patients and was evaluated descriptively due to limited case numbers. **Conclusions**: CLDN18.2 expression may represent a clinically relevant prognostic biomarker reflecting aggressive tumor biology in locally advanced gastric cancer treated with neoadjuvant therapy.

## 1. Introduction

Gastric cancer is the fifth most commonly diagnosed malignancy worldwide and remains one of the leading causes of cancer-related mortality, representing a substantial global health burden [1]. In patients with locally advanced gastric cancer (LAGC), neoadjuvant therapy followed by surgical resection has become the current standard treatment approach [2,3]. Nevertheless, despite advances in perioperative treatment strategies and surgical techniques, pathological response rates and long-term survival outcomes remain suboptimal. These limitations highlight the need for reliable biomarkers capable of predicting treatment outcomes and more accurately reflecting the underlying tumor biology in this patient population [4].

Claudins (CLDNs) are transmembrane proteins located on the apical surface of epithelial cells and constitute essential components of tight junction complexes. They play critical roles in maintaining epithelial cell polarity, intercellular adhesion, migration, and proliferation, while also preserving epithelial barrier integrity and regulating selective paracellular permeability [5].

CLDN18.2 is a claudin isoform expressed in several malignancies, including lung, esophageal, pancreatic, and gastric cancers; however, under physiological conditions, its expression is restricted exclusively to the gastric mucosa [6]. During malignant transformation, disruption of cell polarity results in increased exposure of CLDN18.2 on the tumor cell surface, thereby enhancing its accessibility for therapeutic targeting. These biological characteristics have positioned CLDN18.2 as both an emerging therapeutic target and a clinically relevant biomarker in gastric cancer [6,7].

Recent clinical trials have demonstrated clinical benefit from CLDN18.2-targeted therapies in advanced gastric cancer [8,9]. Reported rates of moderate-to-strong CLDN18.2 expression range from 38.4% to 74.4% [8,10]. However, despite increasing therapeutic interest, the prognostic and predictive significance of CLDN18.2 in locally advanced disease, particularly in patients treated in the neoadjuvant setting, remains insufficiently defined [11]. Given the growing emphasis on biomarker-driven treatment strategies, simultaneous evaluation of additional molecular pathways involved in tumor progression may provide complementary biological insight in this treatment context.

The fibroblast growth factor receptor (FGFR) family comprises transmembrane tyrosine kinase receptors that regulate multiple downstream signaling pathways, particularly the mitogen-activated protein kinase (MAPK) and AKT pathways, which are involved in cellular proliferation, survival, and migration [12]. Activation of FGFR signaling may occur through several genetic mechanisms, including gene amplification, activating mutations, chromosomal rearrangements, or gene fusions. Among these alterations, FGFR2 amplification has been associated with aggressive tumor behavior and unfavorable prognosis in gastric cancer [13].

Previous studies have reported associations between FGFR2 protein overexpression and advanced tumor stage, increased lymph node involvement, and deeper tumor invasion [14]. The prevalence of FGFR2 gene amplification in gastric cancer has been reported to range between 4% and 7% [12,13]. Furthermore, FGFR2b-targeted therapies are currently under active clinical investigation in advanced gastric cancer [15,16]. Accordingly, combined evaluation of FGFR2 and CLDN18.2 expression may contribute to a more comprehensive biological characterization of locally advanced disease.

Therefore, the aim of the present study was to evaluate the prognostic relevance of pretreatment immunohistochemical expression of CLDN18.2 and FGFR2b in patients with locally advanced gastric cancer treated with neoadjuvant therapy.

## 2. Materials and Methods

### 2.1. Study Design and Patient Selection

This retrospective single-center study included 64 consecutive patients diagnosed with LAGC or gastroesophageal junction adenocarcinoma based on pretreatment endoscopic biopsy findings. Patients diagnosed and treated between October 2023 and September 2025 were included in the study. All patients received neoadjuvant therapy followed by curative-intent surgical resection. Pretreatment diagnostic endoscopic biopsy specimens were retrieved from the pathology archives for immunohistochemical (IHC) analysis.

Patients with incomplete clinical data, insufficient biopsy material for IHC evaluation, or those who did not complete neoadjuvant treatment were excluded from the study.

### 2.2. Treatment Protocol

All patients received perioperative chemotherapy consisting of fluorouracil, leucovorin, oxaliplatin, and docetaxel administered prior to surgical resection. Following surgery, adjuvant chemotherapy was planned according to institutional practice and patient tolerance. Minor variations in the number of administered treatment cycles occurred due to clinical or logistical reasons.

None of the patients received concurrent targeted therapies (including anti-HER2 agents) in addition to standard perioperative chemotherapy.

Adjuvant radiotherapy was administered in selected patients based on pathological risk factors and was recorded as a clinicopathological variable for survival analyses.

### 2.3. Pathological Evaluation

All resection specimens were processed according to institutional standard pathology protocols. Tumor staging was performed using the American Joint Committee on Cancer (AJCC) 8th edition Tumor–Node–Metastasis (TNM) classification system.

Histopathological response to neoadjuvant therapy was evaluated on hematoxylin and eosin-stained sections with a thickness of approximately 4 μm. Tumor regression was graded according to the Ryan tumor regression grading (TRG) system as follows: grade 0, complete response with no viable tumor cells; grade 1, near-complete response with minimal residual tumor; grade 2, partial response with residual tumor present; and grade 3, poor or no response with extensive residual tumor [17].

Post-treatment pathological parameters, including post-treatment primary tumor stage (ypT), post-treatment nodal stage (ypN), lymphovascular invasion (LVI), and perineural invasion (PNI), were recorded. Surgical resection margins were classified as negative (R0) or positive (R1/R2). The type of gastric resection (subtotal or total gastrectomy) and the extent of lymphadenectomy (D1 or D2) were documented according to surgical and pathological reports.

Tumor location was classified as proximal (including tumors involving the cardia, fundus, and gastroesophageal junction) or distal (body, antrum, and pylorus) according to endoscopic and surgical findings. D2 lymphadenectomy was performed in accordance with standard Japanese Gastric Cancer Association guidelines.

All histopathological evaluations were performed by a single experienced gastrointestinal pathologist who was blinded to clinical and treatment-related data.

### 2.4. Immunohistochemistry and Scoring

Immunohistochemical staining was performed on formalin-fixed, paraffin-embedded pretreatment endoscopic biopsy specimens using an automated staining platform (Ventana Medical Systems, Roche Diagnostics, Tucson, AZ, USA). Antigen retrieval and staining procedures were performed according to the manufacturers’ protocols. Appropriate positive and negative controls were included in each staining run.

CLDN18.2 expression was assessed using the ZETA Z2807RT antibody (clone ZR451, dilution 1:200). Tumors were classified as CLDN18.2-positive when moderate to strong membranous staining was observed in at least 75% of tumor cells, in accordance with previously reported CLDN18.2-targeted clinical study criteria [8,9].

FGFR2b expression was evaluated using a rabbit polyclonal antibody (HUABIO (Woburn, MA, USA), R1311-7, dilution 1:100). FGFR2b overexpression was defined as moderate to strong membranous staining in more than 0% of tumor cells, consistent with previously published studies [15,16].

Normal gastric mucosa served as the positive control for CLDN18.2 staining, while previously confirmed FGFR2b-positive tumor tissue served as the positive control for FGFR2b.

All IHC slides were evaluated by the same experienced gastrointestinal pathologist who was blinded to clinical and treatment-related data. Staining intensity and distribution were assessed, and classification was performed according to predefined cutoff values derived from the literature.

To minimize the potential impact of intratumoral heterogeneity, all available tumor-containing biopsy fragments were evaluated, and staining assessment was based on overall membranous expression across tumor areas.

### 2.5. Statistical Analysis

Descriptive statistics were used to summarize study variables. Continuous variables were presented as mean ± standard deviation or median (minimum–maximum), as appropriate, while categorical variables were expressed as frequency (percentage). Normality of distribution was assessed using the Kolmogorov–Smirnov and Shapiro–Wilk tests.

Relapse-free survival (RFS) was defined as the time from surgical resection to the first documented recurrence or death from any cause, and overall survival (OS) was defined as the time from the date of curative surgical resection to death from any cause. Survival curves were estimated using the Kaplan–Meier method and compared using the log-rank test.

Associations between clinicopathological variables and survival outcomes were evaluated using univariable and multivariable Cox proportional hazards regression analyses. Variables with *p* values < 0.10 in univariable analysis were entered into the multivariable Cox regression model using the forward likelihood ratio method. Patients without an event were censored at the date of last follow-up. The proportional hazards assumption was assessed. Multicollinearity among covariates included in the multivariable models was assessed using variance inflation factors (VIFs), and no significant collinearity was detected (all VIFs < 3).

Exploratory subgroup analyses were performed to evaluate the prognostic impact of CLDN18.2 expression across predefined clinicopathological subgroups.

All statistical analyses were performed using SPSS Statistics for Windows, version 28.0 (IBM Corp., Armonk, NY, USA). A two-sided *p* value < 0.05 was considered statistically significant.

## 3. Results

### 3.1. Patient and Tumor Characteristics

Baseline demographic and clinical characteristics of the study population according to CLDN18.2 expression status are summarized in Table 1. A total of 64 patients were included in the analysis, of whom 19 patients (29.7%) were classified as CLDN18.2-positive and 45 patients (70.3%) as CLDN18.2-negative.

Patient age distribution and sex were comparable between CLDN18.2-positive and CLDN18.2-negative groups (all *p* > 0.05). Similarly, the prevalence of comorbid conditions, including diabetes mellitus, hypertension, hyperlipidemia, and coronary artery disease, did not significantly differ according to CLDN18.2 expression status.

Baseline tumor characteristics at diagnosis were balanced between groups. Tumor localization, clinical primary tumor stage (cT), clinical nodal stage (cN), and overall clinical stage according to the AJCC 8th edition showed no statistically significant association with CLDN18.2 expression (all *p* > 0.05). Histopathological features evaluated in pretreatment biopsy specimens, including histological subtype, presence of a signet-ring cell component, tumor differentiation grade, and microsatellite instability status, were similarly distributed between CLDN18.2-positive and CLDN18.2-negative patients.

FGFR2b positivity was identified in 9 patients (14.1%). Owing to the limited number of FGFR2b-positive cases, comparative analyses were not performed. Clinicopathological characteristics of FGFR2b-positive patients are presented descriptively in Table 1, and no cases of concurrent CLDN18.2 and FGFR2b positivity were observed.

**Figure 1:**
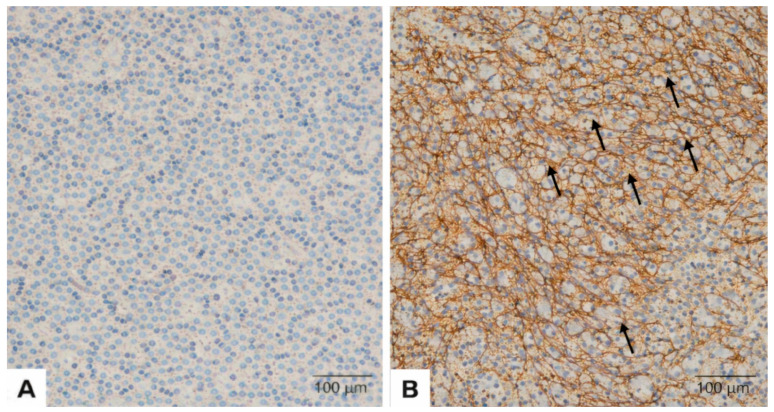
Representative immunohistochemical staining patterns of FGFR2b in pretreatment tumor biopsy specimens. Black arrows indicate membranous staining in tumor cells. (**A**) Absence of membranous FGFR2b staining in tumor cells. (**B**) Moderate to strong membranous FGFR2b positivity consistent with FGFR2b overexpression. Original magnification: ×20.

Representative immunohistochemical staining patterns of FGFR2 are shown in Figure 1.

### 3.2. Surgical and Post-Treatment Pathological Findings According to CLDN18.2 Expression

Surgical and post-treatment pathological characteristics stratified according to CLDN18.2 expression status are summarized in Table 2.

No statistically significant differences were observed between CLDN18.2-positive and CLDN18.2-negative patients with respect to surgical procedures, including type of gastrectomy and extent of lymphadenectomy (all *p* > 0.05). Resection margin status was also comparable between groups.

Post-treatment pathological findings demonstrated similar distributions of pathological tumor depth (ypT stage), nodal involvement (ypN stage), and overall pathological stage between CLDN18.2-positive and CLDN18.2-negative tumors (all *p* > 0.05).

Treatment response assessed according to the Ryan tumor regression grading (TRG) system did not significantly differ according to CLDN18.2 expression status. The frequencies of lymphovascular invasion and perineural invasion were also comparable between groups, and no cases of complete pathological response (TRG grade 0) were observed.

Administration of adjuvant radiotherapy showed no significant association with CLDN18.2 expression (*p* > 0.05).

**Figure 2:**
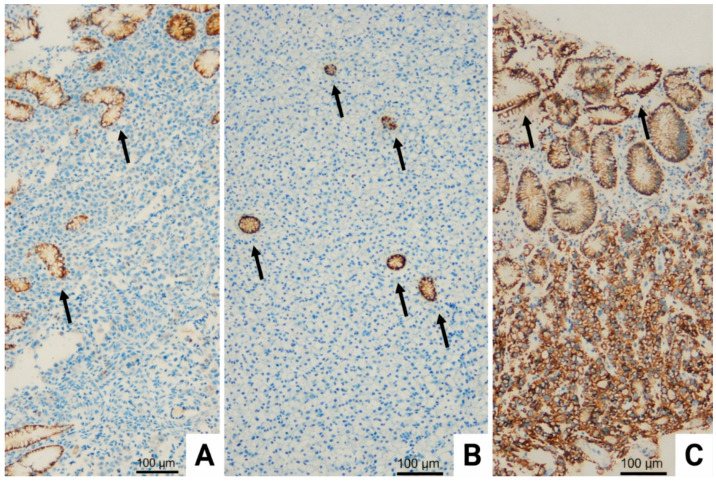
Representative immunohistochemical staining patterns of CLDN18.2 in pretreatment gastric cancer biopsy specimens. Black arrows indicate membranous staining in surface epithelium and entrapped glands serving as internal positive controls. (**A**) Absence of CLDN18.2 expression in poorly differentiated adenocarcinoma cells. (**B**) Negative CLDN18.2 staining in signet-ring cell carcinoma. (**C**) Strong membranous CLDN18.2 positivity observed in ≥75% of tumor cells in poorly differentiated adenocarcinoma. Original magnification: ×20.

Representative immunohistochemical staining patterns of CLDN18.2 are shown in Figure 2.

Overall, CLDN18.2 expression was not associated with surgical or post-treatment pathological characteristics in this cohort.

### 3.3. Survival Analyses According to CLDN18.2 Expression

#### 3.3.1. Relapse-Free Survival (RFS)

**Figure 3:**
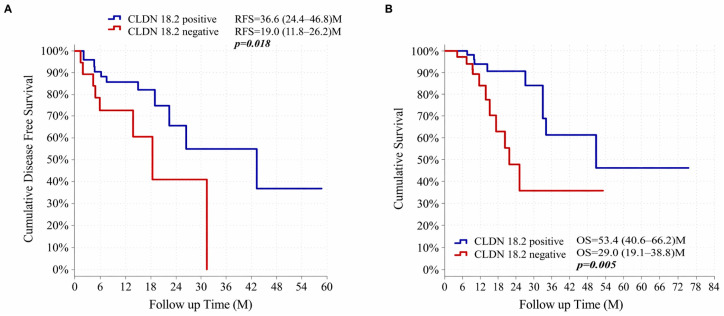
Kaplan–Meier survival curves according to Claudin-18.2 expression status. (**A**) Kaplan–Meier curves for relapse-free survival (RFS) stratified according to Claudin-18.2 (CLDN18.2) expression status. Patients with CLDN18.2-positive tumors demonstrated significantly shorter RFS compared with CLDN18.2-negative patients (median RFS: 19.0 months [95% CI: 11.8–26.2] vs. 36.6 months [95% CI: 26.4–46.8]; log-rank *p* = 0.038). (**B**) Kaplan–Meier curves for overall survival (OS) according to CLDN18.2 expression status. CLDN18.2-positive patients showed significantly shorter OS compared with CLDN18.2-negative patients (median OS: 28.9 months [95% CI: 19.1–38.8] vs. 53.4 months [95% CI: 40.6–66.2]; log-rank *p* = 0.005). Survival distributions were compared using the log-rank test.

The median follow-up duration was 18.8 months (range: 3.8–78.3), with a mean follow-up of 22.2 months. RFS and OS were evaluated according to CLDN18.2 expression status using the Kaplan–Meier method, and survival distributions were compared using the log-rank test (Figure 3).

Patients with CLDN18.2-positive tumors demonstrated significantly shorter RFS compared with CLDN18.2-negative patients. Median RFS was 19.0 months (95% confidence interval [CI]: 11.8–26.2) in the CLDN18.2-positive group and 36.6 months (95% CI: 26.4–46.8) in the CLDN18.2-negative group (log-rank *p* = 0.038) (Figure 3A).

To further evaluate prognostic factors associated with relapse, Cox proportional hazards regression analyses were performed (Table 3). In univariable analysis, CLDN18.2 positivity was significantly associated with poorer RFS (hazard ratio [HR] = 2.618, 95% CI: 1.018–6.735, *p* = 0.046). Clinical nodal stage, post-treatment nodal stage, and lymphovascular invasion were also significantly associated with decreased RFS.

#### 3.3.2. Overall Survival (OS)

OS was analyzed using the Kaplan–Meier method according to CLDN18.2 expression status (Figure 3B). Patients with CLDN18.2-positive tumors demonstrated significantly shorter OS compared with CLDN18.2-negative patients (log-rank *p* = 0.005). Median OS was 28.9 months (95% confidence interval [CI]: 19.1–38.8) in the CLDN18.2-positive group and 53.4 months (95% CI: 40.6–66.2) in the CLDN18.2-negative group.

To further evaluate prognostic factors associated with OS, Cox proportional hazards regression analyses were performed (Table 4). In univariable analysis, clinical nodal stage, post-treatment tumor stage, post-treatment nodal stage, pathological stage, lymphovascular invasion, and administration of adjuvant radiotherapy were significantly associated with OS. Although CLDN18.2 positivity was associated with worse survival in univariable analysis, this association did not remain statistically significant after adjustment for clinicopathological variables in multivariable analysis. Clinical nodal stage and lymphovascular invasion remained independent predictors of OS.

## 4. Discussion

In this study, we investigated the clinical relevance of pretreatment immunohistochemical expression of CLDN18.2 and FGFR2b in patients with locally advanced gastric cancer treated with neoadjuvant therapy. The principal finding of our study is that CLDN18.2 positivity was associated with inferior survival outcomes, remaining an independent predictor of relapse-free survival, while its association with overall survival did not persist after multivariable adjustment. Specifically, CLDN18.2-positive tumors demonstrated shorter relapse-free survival and overall survival, while CLDN18.2 expression remained an independent predictor of relapse after adjustment for established clinicopathological prognostic factors. Importantly, CLDN18.2 expression was not associated with baseline clinical characteristics or pathological treatment response parameters, suggesting that its prognostic relevance may primarily reflect intrinsic tumor biology rather than treatment sensitivity.

Consistent with established evidence, classical prognostic factors retained their significance in our cohort. Advanced clinical lymph node involvement and lymphovascular invasion were independently associated with adverse survival outcomes, supporting the prognostic validity of nodal staging within the 8th edition Tumor–Node–Metastasis classification system and prior reports evaluating patients undergoing surgery following neoadjuvant therapy [18,19,20]. The association between adjuvant radiotherapy and overall survival observed in univariable analysis likely reflects confounding by indication, as radiotherapy is preferentially administered in patients with adverse pathological features.

Notably, CLDN18.2 expression was not associated with tumor regression grade assessed using the Ryan classification. Instead, CLDN18.2 demonstrated a stronger relationship with long-term survival outcomes than pathological response parameters. These findings suggest that CLDN18.2 may capture biological aggressiveness that is not adequately reflected by short-term treatment response metrics. Similar observations have been reported in studies demonstrating heterogeneous prognostic performance of tumor regression grading systems in gastric cancer treated with neoadjuvant therapy [21,22].

Interest in CLDN18.2 has increased substantially following the development of CLDN18.2-targeted therapies; however, data regarding its prognostic role in the neoadjuvant setting remain limited [23]. While some studies have not demonstrated associations between CLDN18.2 expression and treatment response or survival outcomes, variability in disease stage distribution, assessment methodology, and follow-up duration may account for discrepant findings across cohorts. Our results provide additional evidence supporting a potential association between CLDN18.2 positivity and an unfavorable clinical course in locally advanced disease.

From a biological perspective, CLDN18.2 is a stomach-specific tight junction protein that becomes increasingly exposed on tumor cell membranes following disruption of epithelial polarity during malignant transformation [24]. Emerging molecular analyses suggest that CLDN18.2 overexpression is associated with alterations in cell–cell adhesion pathways, cytoskeletal remodeling, immune microenvironment modulation, and activation of pro-survival signaling networks that may collectively promote tumor progression and metastatic potential [25]. These mechanisms provide a plausible explanation for the adverse survival outcomes observed among CLDN18.2-positive patients in our cohort.

FGFR2b positivity was detected in a limited subset of patients, consistent with previously reported prevalence rates [26]. Owing to the small number of FGFR2b-positive tumors, prognostic analyses were restricted to descriptive evaluation. Intratumoral heterogeneity and sampling limitations inherent to endoscopic biopsy specimens may further contribute to underestimation of FGFR2b alterations [27]. In line with large cohort analyses demonstrating rare co-expression of CLDN18.2 and FGFR2b [28], no concurrent positivity was observed in our study population. Accordingly, although FGFR2b expression was included as part of the initial biomarker assessment strategy, the limited number of positive cases restricted its evaluation to descriptive analyses, and these findings should therefore be interpreted as exploratory.

Several limitations should be acknowledged. The retrospective design and relatively limited sample size may restrict generalizability. Although treatment exposure was largely homogeneous, incorporation of adjuvant radiotherapy and the low prevalence of FGFR2b expression limited subgroup analyses. In addition, FGFR2b status was assessed solely by immunohistochemistry without systematic genomic confirmation, and therefore FGFR2b-related findings should be interpreted as exploratory. Prospective validation in larger cohorts using standardized assessment methodologies is warranted.

Collectively, our findings suggest that assessment of CLDN18.2 expression in pretreatment biopsy specimens may contribute to improved risk stratification in patients with locally advanced gastric cancer undergoing neoadjuvant therapy. The integration of biologically relevant biomarkers alongside conventional clinicopathological parameters may enhance prognostic assessment and facilitate the development of future biomarker-guided therapeutic strategies. Despite the relatively limited sample size, the biological consistency of the observed survival association, together with the use of predefined immunohistochemical assessment criteria, supports the internal validity of the present findings.

## 5. Conclusions

In conclusion, CLDN18.2 positivity was independently associated with poorer relapse-free survival and significantly associated with inferior overall survival in patients with locally advanced gastric cancer treated with neoadjuvant therapy. In contrast, FGFR2b expression was infrequent and therefore could only be evaluated descriptively, and these findings should be interpreted as exploratory. These findings support the potential role of CLDN18.2 as a clinically relevant prognostic biomarker in the neoadjuvant setting and warrant prospective validation.

## Figures and Tables

**Table 1:** Baseline clinicopathological characteristics of the study cohort according to FGFR2b and CLDN18.2 expression.

Variables	All (*N* = 64)	FGFR2b Positive (*N* = 9)	CLDN18.2 Positive (*N* = 19)	CLDN18.2 Negative (*N* = 45)	*p* Value
**Age**					0.813
◦ <65 ◦ ≥65	39 (61) 25 (39)	3 (33.3) 6 (66.6)	12 (63.2) 7 (36.8)	27 (60) 18 (40)	
**Gender**					0.834
◦ Female ◦ Male	46 (71.9) 18 (28.1)	5 (55.6) 4 (44.4)	14 (73.7) 5 (26.3)	32 (71.1) 13 (28.9)	
**Comorbidities**					
◦ Diabetes mellitus ◦ Hypertension ◦ Hyperlipidemia ◦ Coronary artery disease	15 (23.4) 29 (45.3) 13 (20.3) 10 (15.6)	3 (33.3) 4 (44.4) 2 (22.2) 3 (33.3)	5 (26.3) 7 (36.8) 3 (15.8) 2 (10.5)	10 (22.2) 22 (48.9) 10 (22.2) 8 (17.8)	0.753 0.376 0.559 0.465
**Tumor Location**					0.117
◦ Proximal ◦ Distal	40 (62.5) 24 (37.5)	7 (77.8) 2 (22.2)	11 (57.9) 8 (42.1)	29 (64.4) 16 (35.6)	
**Clinical T stage**					0.616
◦ T2 ◦ T3 ◦ T4	8 (12.5) 30 (46.9) 26 (40.6)	1 (11.1) 4 (44.4) 4 (44.4)	3 (15.8) 10 (52.6) 6 (31.6)	5 (11.1) 20 (44.4) 20 (44.4)	
**Clinical N stage**					0.801
◦ N0 ◦ N1 ◦ N2 ◦ N3	11 (17.2) 26 (40.6) 16 (25.0) 11 (17.2)	2 (22.2) 5 (55.6) 0 2 (22.2)	2 (10.5) 8 (42.1) 6 (31.6) 3 (15.8)	9 (20) 18 (40) 10 (22.2) 8 (15.8)	
**Clinical Stage (AJCC 8th)**					1.000
◦ II ◦ III ◦ IV	17 (26.6) 38 (59.4) 9 (14.1)	3 (33.3) 5 (55.6) 1 (11.1)	5 (26.3) 11 (57.9) 3 (15.8)	12 (26.6) 27 (60) 6 (13.3)	
**Histology (WHO)**					0.513
◦ Adenocarcinoma ◦ Signet-ring cell ◦ Mucinous ◦ Others	46 (71.9) 12 (18.8) 5 (7.8) 1 (1.6)	8 (88.9) 0 1 (11.1) 0	12 (63.2) 5 (26.3) 1 (5.3) 1 (5.3)	34 (75.6) 7 (15.6) 4 (8.9) 0	
**Signet-Ring Cell Component**					0.695
◦ Absent ◦ Present	28 (43.8) 30 (46.9)	7 (77.8) 2 (22.2)	8 (42.1) 10 (52.6)	20 (44.4) 20 (44.4)	
**Tumor grade**					0.420
◦ G2 (moderately differentiated) ◦ G3 (poorly differentiated)	9 (14.1) 30 (46.9)	0 5 (100)	4 (21.1) 9 (47.4)	5 (11.1) 21 (46.7)	
**MSI status**					1.000
◦ MSS ◦ MSI-H	59 (92.2) 1 (1.6)	9 (100) 0	17 (89.5) 0	42 (93.3) 1 (4.4)	
**HER-2 status**					0.282
◦ Absent ◦ Present	63 (98.4) 1 (1.6)	9 (100) 0	18 (94.7) 1 (5.3)	45 (100) 0	

Values are presented as number (%). Categorical variables were compared using the χ^2^ test or Fisher’s exact test, as appropriate. Analyses comparing clinicopathological variables were primarily performed according to CLDN18.2 expression status. Due to missing data for selected clinicopathological variables, analyses were conducted using available-case methodology; therefore, denominators may vary across variables presented in the table. Given the limited number of FGFR2b-positive tumors, results related to FGFR2b expression should be interpreted descriptively, and no definitive statistical conclusions were intended for this subgroup. Missing data were present for tumor grade (*n* = 25), MSI status (*n* = 4), signet-ring cell component (*n* = 6), and smoking history (*n* = 3). Variable names are presented in bold. Abbreviations: AJCC, American Joint Committee on Cancer; WHO, World Health Organization; MSS, microsatellite stable; MSI-H, microsatellite instability–high.

**Table 2:** Postoperative pathological characteristics and treatment features according to FGFR2b and CLDN18.2 expression.

Variables	All (*N* = 64)	FGFR2b Positive (*N* = 9)	CLDN18.2 Positive (*N* = 19)	CLDN18.2 Negative (*N* = 45)	*p* Value
**Type of gastrectomy**					0.113
◦ Subtotal ◦ Total	15 (23.4) 49 (76.6)	4 (44.4) 5 (55.6)	2 (10.5) 17 (89.5)	13 (28.9) 32 (71.1)	
**Extent of lymphadenectomy**					0.847
◦ D1 ◦ D2	11 (17.2) 53 (82.8)	1 (11.1) 8 (88.9)	3 (15.8) 16 (84.2)	8 (17.8) 37 (82.2)	
**Resection margin**					0.796
◦ R0 ◦ R1	55 (85.9) 9 (14.1)	8 (88.9) 1 (11.1)	16 (84.2) 3 (15.8)	39 (86.7) 6 (13.3)	
**ypT stage**					0.975
◦ T1 ◦ T2 ◦ T3 ◦ T4	10 (15.6) 8 (12.5) 14 (21.9) 32 (50.0)	2 (22.2) 1 (11.1) 2 (22.2) 4 (44.4)	3 (15.8) 3 (15.8) 4 (21.1) 9 (47.4)	7 (15.6) 5 (11.1) 10 (22.2) 23 (51.1)	
**ypN stage**					0.957
◦ N0 ◦ N1 ◦ N2 ◦ N3	16 (25.0) 8 (12.5) 12 (18.8) 28 (43.8)	3 (33.3) 2 (22.2) 0 4 (44.4)	4 (21.1) 2 (10.5) 4 (21.1) 9 (47.4)	12 (26.7) 6 (13.3) 8 (17.8) 19 (42.1)	
**yp Stage (AJCC 8th)**					0.728
◦ I ◦ II ◦ III	12 (18.8) 11 (17.2) 41 (64.1)	2 (22.2) 2 (22.2) 5 (55.6)	4 (21.1) 2 (10.5) 13 (68.4)	8 (17.8) 9 (20) 28 (62.2)	
**TRG (Ryan)**					0.746
◦ TRG1 ◦ TRG2 ◦ TRG3	14 (21.9) 25 (39.1) 25 (39.1)	3 (33.3) 3 (33.3) 3 (33.3)	4 (21.1) 7 (36.8) 8 (42.1)	10 (22.2) 18 (40) 17 (37.8)	
**Lymphovascular invasion**					0.876
◦ Negative ◦ Positive	26 (40.9) 38 (59.4)	4 (44.4) 5 (55.6)	8 (42.1) 11 (57.9)	18 (40) 27 (60)	
**Perineural invasion**					0.750
◦ Negative ◦ Positive	34 (53.1) 30 (46.9)	5 (55.6) 4 (44.4)	10 (52.6) 8 (42.1)	23 (51.1) 22 (48.9)	
**Adjuvant radiotherapy**					0.724
◦ No ◦ Yes	49 (76.6) 15 (23.4)	8 (88.9) 1 (11.1)	14 (73.7) 5 (26.3)	35 (77.8) 10 (22.2)	

Values are presented as number (%). Categorical variables were compared using the χ^2^ test or Fisher’s exact test, as appropriate. Comparisons were primarily performed according to CLDN18.2 expression status. Given the limited number of FGFR2b-positive tumors, findings related to FGFR2b expression should be interpreted descriptively. Analyses were conducted using available-case methodology; therefore, denominators may vary across variables where data were missing. Variable names are presented in bold. Abbreviations: TRG, tumor regression grade (Ryan classification); ypT, post-treatment pathological tumor stage; ypN, post-treatment pathological nodal stage; AJCC, American Joint Committee on Cancer.

**Table 3:** Cox proportional hazards regression analysis for relapse-free survival (RFS).

Variables	Univariate Analysis					Multivariate Analysis				
	HR	95% CI			*p*	HR	95% CI			*p*
**Age**	0.990	0.942	-	1.041	0.697					
**Gender**	1.693	0.633	-	4.526	0.294					
**Smoking history**	0.453	0.197	-	1.041	0.062					
**Comorbidities**	1.323	0.514	-	3.402	0.562					
**CLDN18.2 status**	2.618	1.018		6.735	**0.046**	3.766	1.368	-	10.368	**0.010**
**cT stage**	0.724	0.349	-	1.502	0.386					
**cN stage**	2.679	1.511	-	4.752	**0.001**	2.761	1.550	-	4.918	**0.001**
**Clinical stage**	0.980	0.561	-	1.711	0.942					
**Histology**	1.539	0.827	-	2.864	0.173					
**Signet-ring cell component**	1.642	0.545	-	4.948	0.378					
**Tumor grade**	1.904	0.416	-	8.713	0.407					
**Tumor location**	0.889	0.642	-	1.232	0.480					
**Extent of lymphadenectomy**	1.070	0.353	-	3.247	0.905					
**Type of gastrectomy**	0.698	0.247	-	1.974	0.498					
**ypT stage**	1.241	0.790	-	1.950	0.348					
**ypN stage**	1.723	1.071	-	2.772	**0.025**					
**yp stage**	1.661	0.789	-	3.498	0.181					
**Tumor regression grade**	1.526	0.822	-	2.834	0.181					
**MSI status**	0.757	0.091	-	6.314	0.797					
**HER2 status**	0.130	0.001	-	23.31	0.441					
**Resection margin**	1.337	0.424	-	4.212	0.620					
**Lymphovascular invasion**	3.649	1.057	-	12.59	**0.041**	3.492	1.000	-	12.20	**0.050**
**Perineural invasion**	2.331	0.830	-	6.543	0.108					
**Adjuvant radiotherapy**	1.806	0.709	-	4.602	0.216					

Univariable and multivariable Cox proportional hazards regression analyses were performed to identify clinicopathological factors associated with relapse-free survival. Variables with *p* values < 0.10 in univariable analysis were entered into the multivariable model using a forward likelihood ratio method. CLDN18.2 positivity, clinical tumor stage, and lymphovascular invasion remained independently associated with shorter RFS in multivariable analysis. HR, hazard ratio; CI, confidence interval; RFS, relapse-free survival. Variables demonstrating statistical significance in univariable analysis were subsequently entered into a multivariable Cox proportional hazards model. In multivariable analysis, CLDN18.2 positivity remained an independent predictor of relapse-free survival (HR = 3.766, 95% CI: 1.368–10.368, *p* = 0.010), together with clinical tumor stage (HR = 2.761, 95% CI: 1.550–4.918, *p* = 0.001) and lymphovascular invasion (HR = 3.492, 95% CI: 1.000–12.20, *p* = 0.050). These findings indicate that CLDN18.2 positivity was associated with a more than threefold increased risk of relapse. Variable names and statistically significant *p* values (*p* < 0.05) are presented in bold.

**Table 4:** Cox proportional hazards regression analysis for overall survival (OS).

Variables	Univariate Analysis					Multivariate Analysis				
	HR	95% CI			*p*	HR	95% CI			*p*
**Age**	1.016	0.964	-	1.072	0.548					
**Gender**	2.768	1.063	-	7.208	**0.037**					
**Smoking history**	0.831	0.395	-	1.748	0.625					
**Comorbidities**	2.333	0.871	-	6.248	0.265					
**CLDN18.2 status**	3.618	1.394	-	9.396	0.092					
**cT stage**	1.018	0.497	-	2.086	0.960					
**cN stage**	2.085	1.159	-	3.751	**0.014**	1.812	1.022	-	3.214	**0.042**
**Clinical stage**	1.236	0.687	-	2.223	0.479					
**Histology**	1.030	0.509	-	2.084	0.934					
**Signet-ring cell component**	1.408	0.534	-	3.715	0.489					
**Tumor grade**	1.149	0.309	-	4.275	0.836					
**Tumor location**	0.890	0.655	-	1.209	0.456					
**Extent of lymphadenectomy**	1.106	0.318	-	3.843	0.874					
**Type of gastrectomy**	1.694	0.383	-	7.487	0.487					
**ypT stage**	2.029	1.081	-	3.807	**0.028**					
**ypN stage**	2.005	1.156	-	3.476	**0.013**					
**yp stage**	3.975	1.134	-	13.933	**0.031**					
**Tumor regression grade**	1.829	0.946	-	3.536	0.073					
**MSI status**	1.274	0.159	-	10.223	0.820					
**HER2 status**	0.118	0.000	-	89.779	0.528					
**Resection margin**	1.392	0.400	-	4.848	0.604					
**Lymphovascular invasion**	6.499	1.490	-	28.358	**0.013**	5.661	1.273	-	25.179	**0.023**
**Perineural invasion**	2.557	0.933	-	7.009	0.068					
**Adjuvant radiotherapy**	2.899	1.149	-	7.316	**0.024**					
**Recurrence**	2.045	0.806	-	5.184	0.132					

Univariable and multivariable Cox proportional hazards regression analyses were performed to identify clinicopathological factors associated with overall survival. Variables with *p* values < 0.10 in univariable analysis were entered into the multivariable model using a forward likelihood ratio method. HR, hazard ratio; CI, confidence interval; OS, overall survival. Variable names and statistically significant *p* values (*p* < 0.05) are presented in bold.

## Data Availability

The datasets generated and analyzed during the current study are not publicly available due to patient privacy and institutional regulations but are available from the corresponding author on reasonable request.

## Abbreviations

The following abbreviations are used in this manuscript:

**Abbreviation**	**Definition**
CLDN18.2	Claudin-18.2
FGFR2b	Fibroblast Growth Factor Receptor 2b
LAGC	Locally Advanced Gastric Cancer
IHC	Immunohistochemistry
FFPE	Formalin-Fixed Paraffin-Embedded
TRG	Tumor Regression Grade
AJCC	American Joint Committee on Cancer
LVI	Lymphovascular Invasion
PNI	Perineural Invasion
RFS	Relapse-Free Survival
OS	Overall Survival
HR	Hazard Ratio
CI	Confidence Interval
